# M2-type macrophage nanovesicles regulate the inflammatory response after necrotizing enterocolitis by inducing M1 to M2-like macrophage polarization

**DOI:** 10.3389/fcimb.2025.1664897

**Published:** 2025-10-07

**Authors:** Chang Liu, Jie Gong, Lin Zhang, Yu Wang, YaJun Huang, Rong Ju

**Affiliations:** ^1^ Chengdu Women’s and Children’s Central Hospital, School of Medicine, University of Electronic Science and Technology of China, Chengdu, China; ^2^ College of Life Sciences, Inner Mongolia Agricultural University, Hohhot, China; ^3^ Department of Neonatology, People’s Hospital of Jianyang City, Jianyang, China; ^4^ Department of Neonatology, The Affiliated Chengdu 363 Hospital of Southwest Medical University, Chengdu, China

**Keywords:** necrotizing enterocolitis, inflammatory response, macrophages polarization, nanovesicle, gastrointestinal disorder, polarization

## Abstract

Necrotizing enterocolitis (NEC) is a serious inflammatory gastrointestinal disorder leading to a devastating intestinal inflammatory response, which typically results in severe sepsis and death. Given the imbalance of inflammatory response in the intestine that results in immune dysregulation and further worsens the clinical symptoms of NEC. Macrophages are the primary cells responsible for the early regulation and resolution of intestinal inflammation, therefore our experiments focus on the regulation of the polarization type of macrophages. In this study, we applied a convenient and continuous extrusion system to execute and purify M2NVs from RAW264.7 macrophage cells, then used to interfere with the LPS-induced cell inflammation model and NEC animal model. We discovered that M2NVs could foster the polarization of M1 to M2 macrophages and inhibit inflammatory injury *in vivo*, aligning with the *in vitro* results. Meanwhile, our study also revealed that M2NVs intervention could not only effectively alleviate the intestinal inflammatory environment, but also affect the changes in intestinal metabolism via omics techniques. Overall, the engineering strategy of M2NVs represents a promising approach with great potential for NEC treatment.

## Graphical Abstract

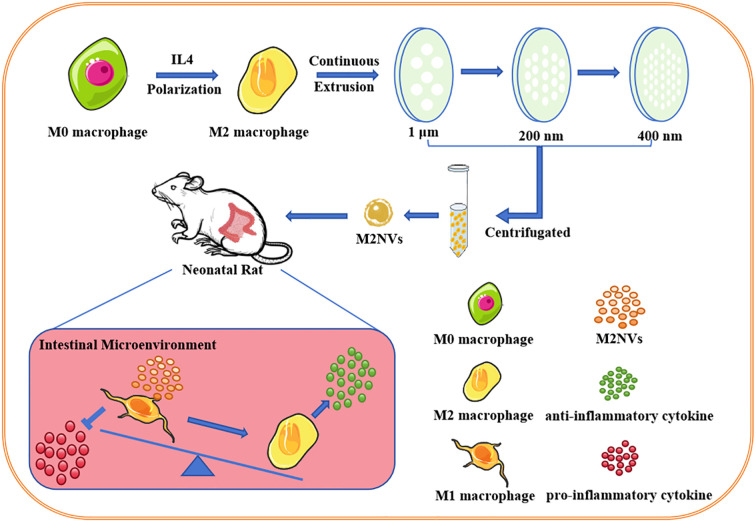

## 1 Introduction

Necrotizing enterocolitis (NEC) is a serious inflammatory gastrointestinal disorder with an incidence ranging from 2% to 13%, primarily affecting premature neonates with a birth weight of less than 1500g. Its symptoms are mostly manifested as ischemic necrosis of the small and large intestines, contributing to a devastating intestinal inflammatory response that typically results in severe sepsis and death (Ou et al., 2020; Kaplina et al., 2023). NEC remains a significant cause of morbidity and mortality (15% to 45%), particularly in premature infants, low birth weight neonates, and newborns receiving enteral feeding (Di et al., 2022). Due to the lack of specific and effective treatments for NEC, current strategies are limited to supportive care, while infants with severe clinical deterioration may require intestinal surgery. However, even those infants who survive surgical treatment for NEC often face severe sequelae, including short bowel syndrome, growth restriction, and neurological dysfunction (Duess et al., 2023; Wei et al., 2023). Therefore, there is an urgent need to find effective therapies to improve its management.

The current understanding of the pathogenesis of NEC is that the excessive pro-inflammatory response and/or insufficient anti-inflammatory response promote an inappropriate inflammatory reaction due to immune dysregulation, further compounded by intestinal barrier injury that worsens the clinical symptoms of NEC (Cho et al., 2020). Macrophages, a key component of the intestinal immune system, are the primary cells responsible for the early regulation and resolution of intestinal inflammation (Pan et al., 2022). Traditionally, macrophages are viewed as highly adaptable cells that rapidly adjust to the tissue microenvironment to fulfill necessary functions. They mainly polarize into two types upon activation: M1 macrophages (‘classically’ activated), which are considered highly pro-inflammatory and arise in response to pro-inflammatory cytokines like IFNγ, and M2 macrophages (‘alternatively’ activated), which are associated with anti-inflammatory responses and tissue repair in response to type 2 inflammatory cytokines, such as IL-4 (Hegarty et al., 2023). Thus, macrophage polarization plays a crucial role in maintaining intestinal homeostasis, and regulating the balance between the M1 and M2 macrophage ratio may enhance the intestinal inflammatory environment (Zhang et al., 2023). However, recent studies suggest that merely increasing the proportion of M2 macrophages, or reprogramming M1 macrophages into M2 macrophages, is not a viable therapeutic strategy, as it not only fails to mitigate the inflammatory damage caused by M1 macrophages but may also worsen the fibrotic reaction (Dharmasiri et al., 2021). Therefore, focusing on effective strategies for manipulating macrophage polarization may present new opportunities for NEC treatment.

In recent years, exosomes membrane-derived vesicles measuring 30–200 nm contain various cellular components, including pro-inflammatory and anti-inflammatory cytokines, proteins, enzymes, and nucleic acids (DNA, RNA, microRNA, non-coding RNAs). Due to their low cytotoxicity, biocompatibility, and low immunogenicity, exosomes have been widely studied as endogenous delivery carriers for disease therapy (Arabpour et al., 2021). Research has demonstrated that M2 macrophages can secrete macrophage-derived exosomes (M2 Exo), which inherit the anti-inflammatory properties of M2 macrophages. These exosomes can deliver active biomolecules that influence gene expression in recipient cells, indicating their potential to treat inflammatory diseases (Li et al., 2022). Despite these promising advantages, the low production of exosomes released from cells and the challenges in collection limit their practical application. Although liposomes (synthetic lipid bilayer vesicles) have been employed as effective substitutes for exosomes, they still lack the innate biocompatibility of endogenous exosomes and are rapidly cleared by the immune system during *in vivo* delivery (Neupane et al., 2023). Recently, cell-derived nanovesicles (NVs), generated from fragmented cellular membranes, have emerged as a promising therapeutic delivery medium due to their high production yield and low immunogenicity when administered *in vivo*.

In conclusion, we developed programmed M2 macrophage-derived nanovesicles to enhance their effectiveness in facilitating the transition of pro-inflammatory macrophages to an anti-inflammatory polarization phenotype. Overall, programmed nanovesicle-based therapy is anticipated to improve the inflammatory cell expression phenotype and regulatory capacity of immune cells, contributing to the treatment of NEC. In our research, we prepared M2NVs through serial extrusion and explored whether these M2NVs could repolarize M1 macrophages into M2 macrophages, thereby reducing the intestinal inflammatory response in NEC, both *in vitro* and *in vivo*. Furthermore, we will establish a comprehensive technical protocol for the preparation and collection of M2NVs, providing safe and feasible nanomaterials for NEC treatment strategies, as well as theoretical and methodological support for their clinical application.

## 2 Materials and methods

### 2.1 Chemicals and reagents

Lipopolysaccharide was purchased from Sigma-Aldrich (St.Louis, MO, USA). Murine IL-4 was obtained from Peprotech Inc. (USA). The IL4, IL6, TNFα, TGFβ ELISA detection kit was gained from MultiSciences (Lianke) Biotech Co., Ltd. (Hangzhou, China). Mini-Extruder set (Avanti Polar Lipids), and Nuclepore polycarbonate Membranes (1.0μm, 200nm, 400nm) were gained from Sigma-Aldrich (St.Louis, MO, USA). Rabbit CD86, CD206, IL6, TGFβ and β-actin antibodies were obtained from Proteintech Group, Inc (Wuhan, China). Mouse IL4 and TNFα antibodies were purchased from Proteintech Group, Inc (Wuhan, China). Horseradish peroxidase (HRP) conjugated secondary antibody was purchased from Proteintech Group.

### 2.2 Macrophage culture and polarization

The murine RAW264.7 was purchased from the Institute of Biochemistry and Cell Biology, Academy of Science (Shanghai, China). Cells were cultured in Dulbecco’s Modified Eagle’s Medium (DMEM) supplemented with 10% fetal bovine serum (FBS), and 100 U/mL penicillin/streptomycin (Thermo Fisher Scientific, USA) at 37°C with 5% CO_2_. The macrophage polarization experiment was designed as follows: RAW264.7 cells were exposed to LPS (100 ng/mL) and IL4 (20 ng/mL) for 24h in a serum-free medium to evaluate the cell morphology and polarization level of macrophage (Zhang et al., 2024).

### 2.3 M2 macrophage nanovesicle preparation and repolarization treatment

M2NVs were produced through a continuous extrusion approach, as described previously (Pang et al., 2023). Firstly, 1×10^6^ macrophage cells were collected and then resuspended in 1mL PBS (pH 7.4) solution and centrifuged (3500xg, 15 min) to remove cells, apoptotic bodies and cell fragments. Secondly, to obtain the M2NVs, the cell suspensions were extruded through a mini-extruder (Avanti 610000-1EA), with a battery of Nuclepore polycarbonate membrane filters (pore sizes: 1.0 μm, 200nm, 400nm, Avanti 610010-1EA, Avanti 610007-1EA, Avanti 610006-1EA),. After the extrusion, the extruded suspension (1mL) was centrifugated at 10000 g for 10 min to purify M2NVs by removing cell debris and micro-vesicles, then, a centrifugal filter was used to concentrate and purify the M2NVs by centrifugation (1000g, 15 min) for three times. Finally, the filtrate was centrifuged at 12000g for 120 min to obtain M2-phenotype exosomes (M2exo), which were resuspended in PBS. All operations were performed under aseptic at 4°C. The macrophage repolarization experiment groups were designed as follows: Control group, LPS-induced M1 macrophage cells group, M2NVs (10^6^/mL) intervention group.

### 2.4 Transmission electron microscopy

To observe the morphology of M2NVs, the precipitation after centrifugation was fixed in 2.5% glutaraldehyde overnight at 4°C, then washed with PBS immersion at least three times before being viewed via transmission electron microscope (JEOL Ltd., Tokyo, Japan).

### 2.5 Nanoparticle tracking analysis

The M2NVs’ particle size, distribution, and concentration were detected via the nanoparticle tracking analysis (NTA) as follows: the M2NVs suspension was diluted to 1 mL volume with distilled water, and then immediately transferred to a 1 mL volume syringe, subsequently, the particle size, distribution and concentration of M2NVs were detected by NTA Nano-Sight NS300 detectors (NanoSight, Malvern, United Kingdom).

### 2.6 Animals and experimental design of NEC

SD rats were obtained from the Experimental Animal Center of Chengdu Dossy Experimental Animals Co., Ltd. (Sichuan, China). All animal experiments were approved by the Institutional Animal Care and Use Committee of Chengdu Women’s and Children’s Central Hospital (protocol number: 2022(145)-192). 3-day-old rat pups were fed by gavage with hyperosmolar formula (every 4 h) [Abbott Nutrition infant formula (USA): Esbilac (PetAg, USA) milk replacer, 2:1]. Rats were simultaneously exposed to at 4°C for 10 min and hypoxic conditions (5% O2, 95% N2) 3 times per day last four days. In addition, to establish the NEC model, the rats were also fed LPS (200μl, 100 ng/mL) daily, and the M2NVs intervention group was given intraperitoneal injection of M2NVs (300μl, 10^6^/mL) once a day until the day before death for building the NEC model. The untreated and age-matched neonatal rats lived with their mothers as the control group. On postnatal day 7, the intestines were harvested for further analysis.

### 2.7 Western blotting

Total proteins from the intestine tissue or RAW264.7 cells were extracted using RIPA buffer added with protease inhibitors. Protein samples (20 μg) were quantified for analysis by BCA protein assay kit. Then, the protein samples were electrophoresed in 8–12% SDS-PAGE gels and transferred to PVDF membranes. After incubation with the respective primary antibodies and corresponding secondary antibodies, the target protein signal was visualized using an enhanced chemiluminescence (ECL) system and analyzed using the Image J software (NIH, Bethesda, MD, USA). The specific protein bands were quantified and normalized to β-actin.

### 2.8 Flow cytometry analysis

After the corresponding treatment, the single-cell suspension made from the cultured cells was incubated with CD86 and CD206 according to the manufacturer’s protocol. The expression level of surface markers of macrophage cells was assessed using flow cytometry analysis using a BD FACS.

### 2.9 Enzyme-linked immunosorbent assay for inflammatory cytokines

Each group of cell culture supernatant was collected and centrifugated to remove the cell debris, and then the concentrations of inflammatory cytokines, including IL-4, IL-6, TNFα, and TGF-β were measured in the purified culture supernatants using commercial ELISA kits according to the manufacturer’s protocols.

### 2.10 Hematoxylin and eosin staining

The intestinal section slices were immersed in hematoxylin for 15 minutes After dewaxing and hydration, and subsequently, immersed in eosin for 7 minutes after washed in distilled water. After being rewashed in distilled water, dehydrated, and mounted. The sections were observed, and digital images were obtained under light microscopy (Olympus). The control group was compared using the slices in equal levels.

### 2.11 Immunofluorescence staining

The tissue slices and cultured cell samples were immersed in 4% paraformaldehyde for 20 min, then blocked in goat serum for 30min and incubated overnight with anti-TGFβ and anti-TNFα (1:200) primary antibody. The next day, cells were incubated for 1h with the corresponding secondary antibody (FITC-green, CY3-red) (1:250). Images were finally generated under fluorescence microscopy (Leica).

### 2.12 Intestinal metabolomics analysis

The weighed intestinal tissue was centrifuged at 14,000 g for 20 min at 4°C after being dried lyophilized and ground for 1 min at 65 Hz in a Grinding Mill and placed for 1 h ultrasonic shaking in ice baths with 1 mL precooled mixtures of methanol, acetonitrile and water (v/v/v, 2:2:1), then the supernatants were recovered and concentrated under vacuum until dry to extract metabolites. The extracted metabolites were analyzed using a UPLC-ESI-Q-Orbitrap-MS system (UHPLC, Shimadzu Nexera X2 LC-30AD, Shimadzu, Japan) coupled with Q-Exactive Plus (Thermo Scientific, San Jose, USA) for metabolomics profiling. For liquid chromatography (LC) separation, the samples were analyzed using a ACQUITY UPLC^®^ HSS T3 column (Waters, Milford, MA, USA) to detect liquid chromatography (LC) separation, the flow rate was 0.3 mL/min and the mobile phase contained: A: 0.1% FA in water and B: 100% acetonitrile (ACN). HESI (electrospray ionization)-MS data was acquired in positive/negative mode over m/z 70-1050, full MS resolution was 70,000 at m/z 200, and 17,500 at m/z 200 for MS/MS scan; MS/MS used a 2 m/z window, stepped NCE (20,30,40), and 17,500 resolution. Models were built on principal component analysis (PCA), the p value was calculated by one-way analysis of variance (ANOVA) for multiple groups analysis, metabolites with VIP values greater than 1.0 and p value less than 0.05 were considered to be statistically significant metabolites. The KEGG pathway analysis was used to identify the perturbed biological pathways via using the KEGG database (http://www.kegg.jp). Finally, multivariate data analysis is used for correlation data pre-processing and filtering.

### 2.13 Statistical analysis

Data are presented as the mean ± SD of at least four independent experiments. All statistical analyses were performed using the SPSS 21.0 software (IBM Corp., Armonk, NY, USA). An unpaired t-test was used for comparison of normal distribution and homogeneous variance samples and differences were determined using one-way analysis of variance (ANOVA) for more than two groups and followed by a Student-Newman-Keuls test for multiple comparisons. *P* < 0.05 or *P* < 0.01 was considered statistically significant.

## 3 Results

### 3.1 Polarization and identification of M1- and M2- macrophages

**Figure 1:**
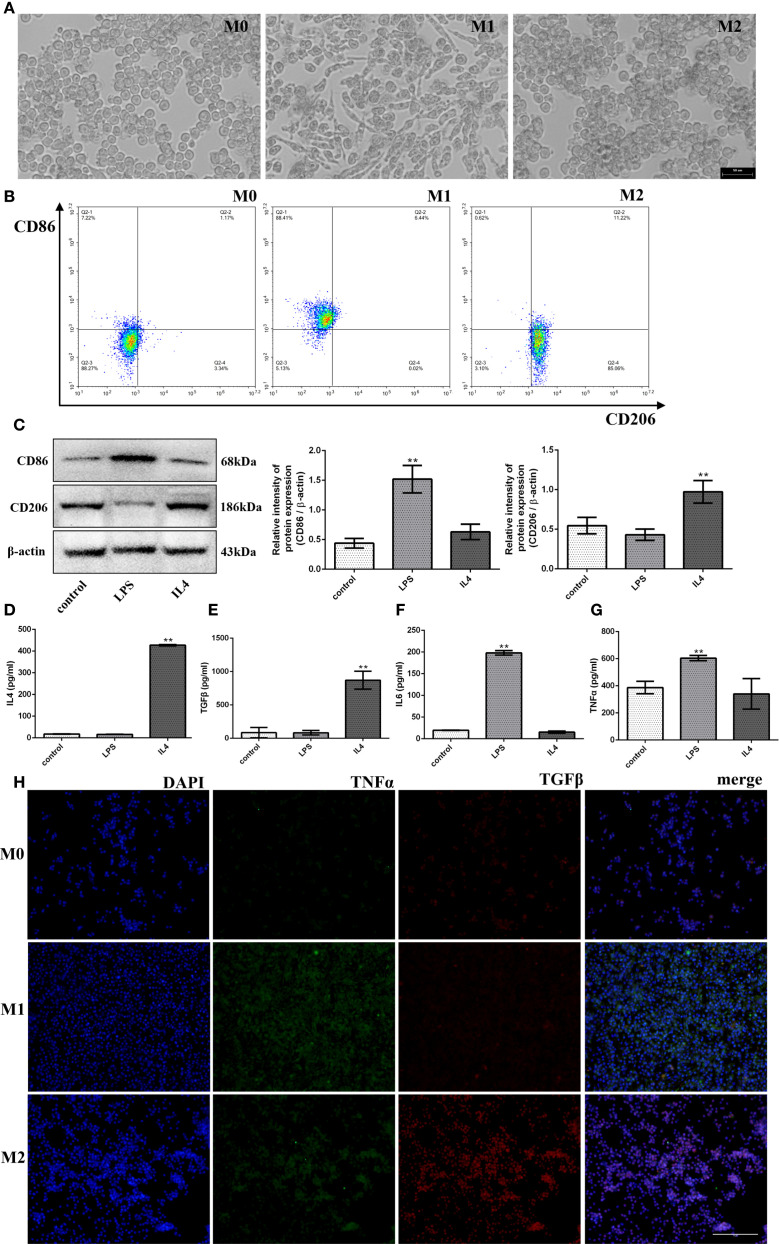
Polarization and identification of M1- and M2- macrophages. After treatment with LPS/IL4. **(A)** The morphology of M0-, M1- and M2-type macrophages under microscope (bar:50μm). **(B)** The detection of CD86 and CD206 (M1- and M2-type macrophages marker) by flow cytometer. **(C)** The protein expression levels of CD86 and CD206, and β-actin are measured using western blot analysis and the relative expression levels are plotted. **(D-G)** The Inflammatory factor expression levels of IL4, TGFβ, IL6 and TNFα are measured using ELISA analysis and the relative expression levels are plotted. **(H)** The fluorescence signal change of TGFβ and TNFα was shown after the treatment of LPS/IL4 (bar:10μm). *p< 0.05 and **p < 0.01 vs control.

Macrophages originating from the mouse mononuclear macrophage cell line RAW264.7 (M0) are a widely established and used system to estimate macrophage polarization mechanisms. Thus, to study the polarization of macrophages *in vitro*, we treated RAW264.7 cells with LPS to polarize into M1-type macrophages and IL4 into M2-type macrophages, which allows us to differentiate cell populations (M0/M1/M2) under the microscope, as shown in **Figure 1A**, under the resting conditions, M0 cells were round and small with clear edges, M1 cells showed the irregular polygonal shape such as spindle-shaped, enlarged or fibrous, whereas, M2 cells were small and rounded like those of M0 cells with low adhesion. In addition, to further verify types of macrophage polarization, we evaluated the expressions of M1 macrophage marker CD86 and M2 macrophage marker CD206 *in vitro* via flow cytometry, results have shown that LPS-induced macrophage polarization mostly clustered in CD86-positive regions (88.41%) and M2-type polarization enhanced by IL4 mostly concentrated in the CD206-positive region (85.06%) (**Figure 1B**). To further verify the type of macrophage polarization, we also conducted western blot experiments to identify the surface markers. We found that the CD86 expression (a marker for M1 macrophages) significantly increased after LPS induction (3.46-fold, *p* < 0.01), while the CD206 expression (a marker for M2 macrophages) was not pronounced. In contrast, the results were reversed (1.78-fold, *p* < 0.01) in the IL4-induced macrophage polarization group, as shown in **Figure 1C**.

Furthermore, we also examined the inflammatory cytokines expression levels in LPS/IL4-induced RAW264.7 cells. As anticipated, we found that the up-regulated expressions of pro-inflammatory cytokines (IL6, TNFα) in the LPS treatment group (*p* < 0.01) and the up-regulated expressions of anti-inflammatory cytokines (IL4, TGFβ) in the IL4 treatment group (*p* < 0.01) compared with the M0 group (**Figures 1D–G**) through ELISA assay. In addition, we also detected the expression of the proinflammatory factor TNFα and the anti-inflammatory factor TGFβ by cellular immunofluorescence assay and found that TNFα expression increased significantly and the expression of TGFβ was not obvious after LPS induction, while the result was opposite after IL4 induction as shown in **Figure 1H**. Taken together, these results implied that macrophage cells could polarize from M0 to M1 or M2, exhibit their characteristics, and release corresponding inflammatory factors.

### 3.2 Preparation, phenotypic identification, and repolarization effect of M2 macrophage nanovesicles *in vitro*

**Figure 2:**
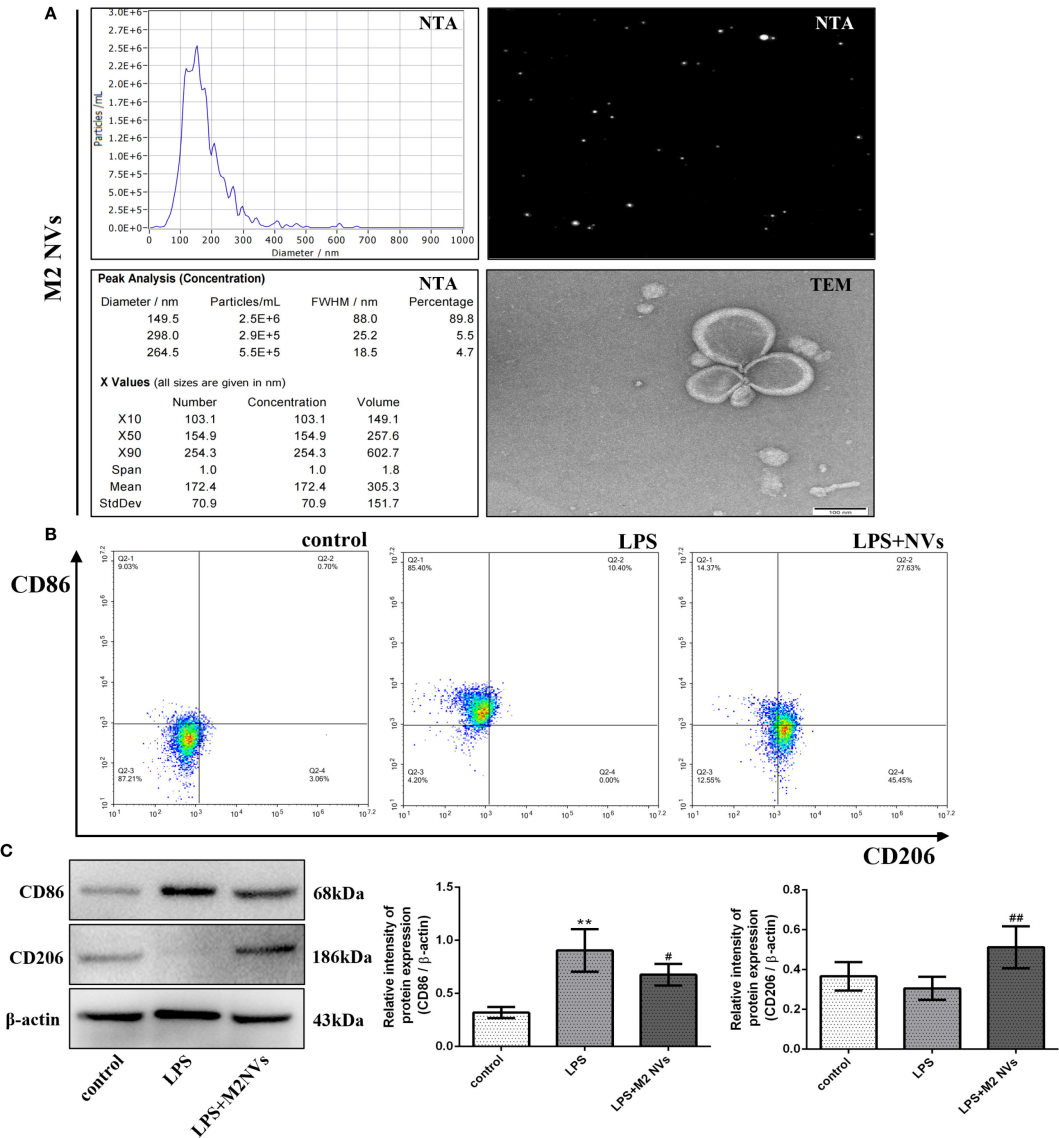
Preparation, phenotypic identification and repolarization effect of M2 macrophage nanovesicles *in vitro*. **(A)** The particle size, distribution and concentration of M2-type macrophages under NTA and TEM detection (bar:100nm). **(B)** The detection of CD86 and CD206 (M1- and M2-type macrophages marker) by flow cytometer. **(C)** The protein expression levels of CD86 and CD206, and β-actin are measured using western blot analysis and the relative expression levels are plotted. *p < 0.05 and **p < 0.01 vs control; ^#^ *p* < 0.05 and ^##^ *p* < 0.01 vs the LPS-treated cells.

To obtain M2NVs, a convenient and continuous extrusion system was established to execute and purify M2NVs from RAW264.7 macrophage cells. To detect the particle size, distribution and concentration, the NTA technique was applied and showed that the M2NVs are regular and granular, the average size of M2NVs was 149.5 nm, the percentage was 89.8%, and the concentration was 2.5*10^6^/mL(**Figure 2A**) which suggested that our method could produce the homogeneous size and moderate concentration of M2NVs. Besides that, TEM images of M2NVs showed that the size of those vesicles ranged from 100 to 150nm and presented as a regular circle with intact spherical membrane structure (**Figure 2A**).

**Figure 3:**
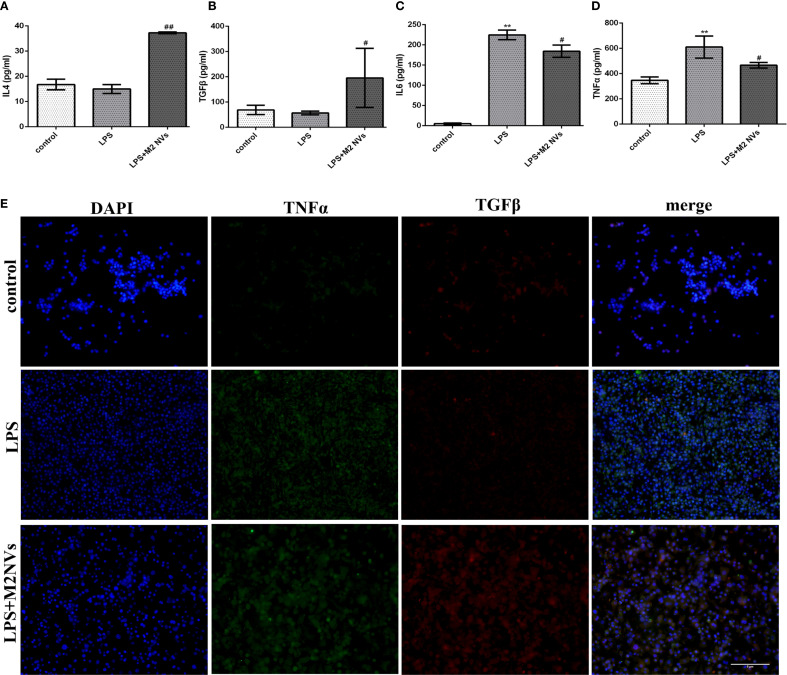
Expression levels of inflammatory factors after treatment of M2NVs. **(A-D)** The Inflammatory factor expression levels of IL4, TGFβ, IL6 and TNFα are measured using ELISA analysis and the relative expression levels are plotted. **(E)** The fluorescence signal change of TGFβ and TNFα was shown after the treatment of M2NVs (bar: 5μm). *p < 0.05 and **p < 0.01 vs control; ^#^ *p* < 0.05 and ^##^ *p* < 0.01 vs the LPS-treated cells.

To further identify whether the M2NVs have an anti-inflammatory function and whether those vesicles could repolarize M1 macrophages into M2 macrophages, the M2NVs were co-cultured with LPS-induced M1 macrophages, then we detected the associated macrophage markers. As shown in **Figures 2B,C**, both flow cytometry and western blot results showed that the M2NVs could alleviate M1 cells’ surface marker CD86 and up-regulate the M2 surface markers CD206 (*p* < 0.01). Subsequently, we further examined the expression levels of inflammatory factors through ELISA assay and demonstrated that M2NVs could mitigate the expressions of pro-inflammatory cytokines (IL6, TNFα) and enhance the expressions of anti-inflammatory cytokines (IL4, TGFβ)(**Figures 3A–D**). Meanwhile, we used cellular immunofluorescence assay to detect the expression of the proinflammatory factor TNFα and the anti-inflammatory factor TGFβ and found that TNFα expression decreased significantly and the expression of TGFβ was up-regulated after M2NVS intervention as shown in **Figure 3E**. Collectively, these results demonstrated that M2NVs could not only repolarize M1 macrophages into M2 macrophages but also have an anti-inflammatory function like M2 macrophage cells.

### 3.3 M2NVs treatment alleviates inflammatory response and injury of NEC *in vivo*

**Figure 4:**
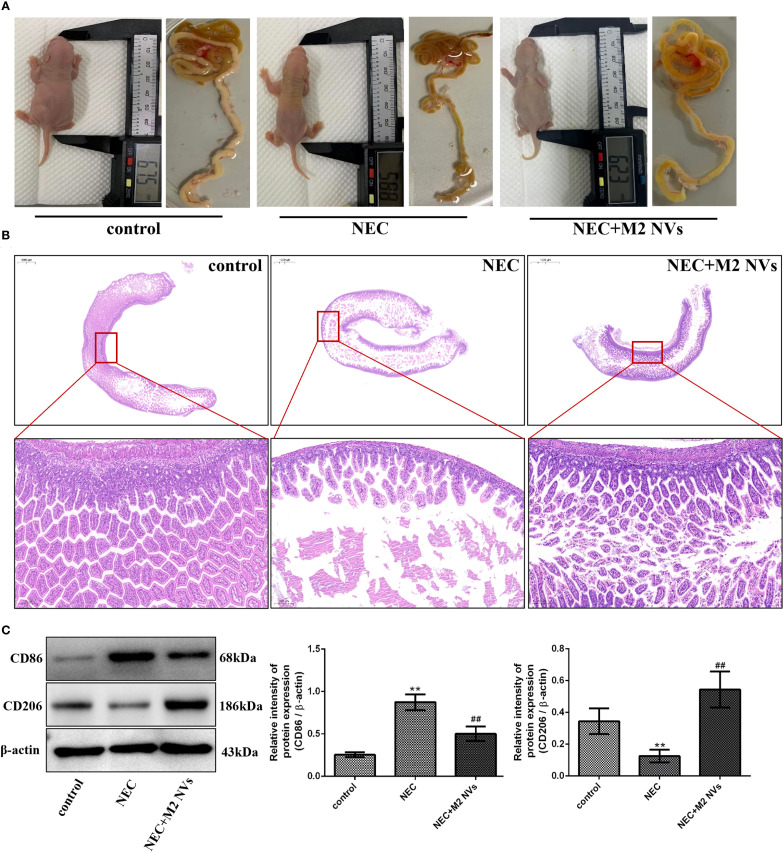
The influence of M2NVs treatment after NEC injury *in vivo*. **(A)** The changes in rats’ size and height and the morphology of intestinal were shown after treatment of M2NVs. **(B)** Histological analyses of the intestinal tissues in WT, NEC rats and M2NVs-treated NEC rats. **(C)** The protein expression levels of CD86 and CD206, and β-actin are measured using western blot analysis and the relative expression levels are plotted. *p < 0.05 and **p < 0.01 vs control; ^#^ *p* < 0.05 and ^##^ *p* < 0.01 vs the NEC-treated group.

To verify whether M2NVs have the function of therapeutic repair effect *in vivo*, we constructed a neonatal rat NEC model. As shown in **Figure 4A**, we found that rats in the control group were well-developed with intact intestinal tissue, and the intestinal tissue was well-elastic and non-congestion with non-bubbles, while in the NEC group, almost all intestinal tissues were black and red, the intestinal tubes were fragile and some intestinal segments were dilated, as expected, intestinal tissue damage was significantly reduced in the M2NVs treatment group, and the intestinal color was close to normal, with only local discoloration with no obvious dilatation on the intestinal wall. In addition, HE staining and pathological examination were used to determine intestinal microscopic changes and showed that the villous intestinal epithelial cells in the NEC group were disordered with submucosal edema and local villi shedding, however, in the M2NVs treatment group, the disorder of villous epithelial, submucosal edema and villi shedding showed improvement (**Figure 4B**).

**Figure 5:**
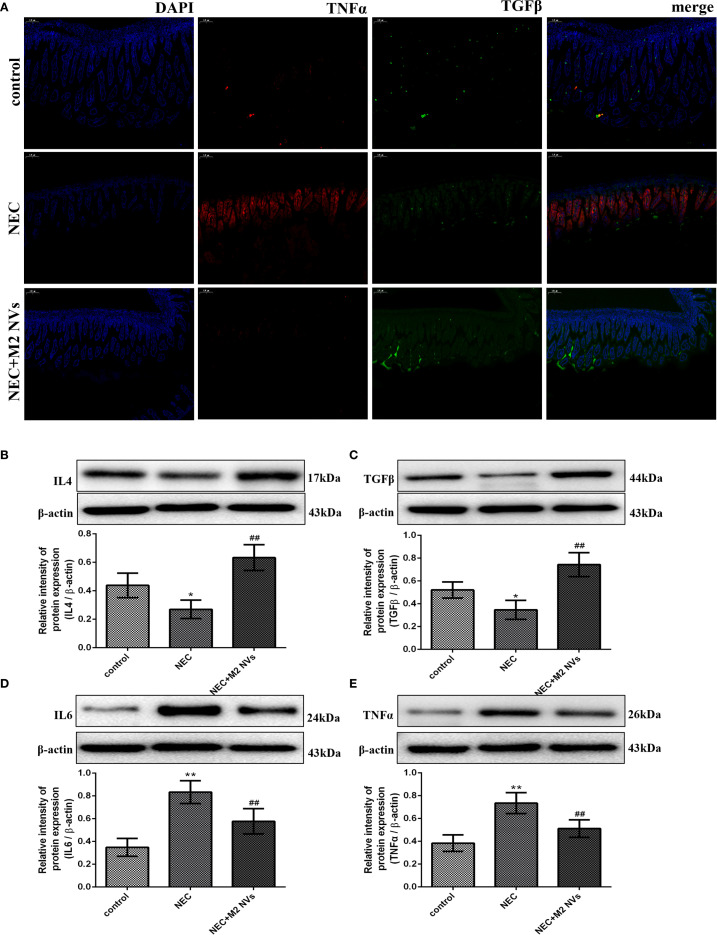
M2NVs treatment alleviates inflammatory response and injury of NEC *in vivo*. **(A)** The fluorescence signal change of TGFβ and TNFα was shown after the treatment of M2NVs. **(B-E)** The Inflammatory factor expression levels of IL4, TGFβ, IL6 and TNFα are measured using western blot analysis and the relative expression levels are plotted. *p < 0.05 and **p < 0.01 vs control; ^#^ *p* < 0.05 and ^##^ *p* < 0.01 vs the NEC-treated group.

Subsequently, we examined whether M2NVs have the function of repolarizing M1 into M2-type macrophages, the M1 cells’ surface marker CD86 and M2 surface markers CD206 were detected, and results showed that M2NVs could reduce CD86 expression effectively (decline rate was 42.5%, *p* < 0.01) and intensify CD206 expression after M2NVs intervention (4.34-fold, *p* < 0.01) (**Figure 4C**). Furthermore, to further verify whether M2NVs have the role in reducing inflammatory symptoms, we also examined the expression of the proinflammatory factor TNFα and the anti-inflammatory factor TGFβ via cellular immunofluorescence assay and found that TNFα expression decreased significantly and the expression of TGFβ was up-regulated after M2NVS intervention as shown in **Figure 5A**. Also, the associated intestinal inflammatory factors were determined by ELISA kit and found that M2NVs intervention alleviated the pro-inflammatory cytokines expressions (IL6, TNFα) and strengthened the anti-inflammatory cytokines expressions (IL4, TGFβ) *in vivo* (*p* < 0.01, **Figures 5B–E**). These data also confirmed that M2NVs could effectively repolarize M1 into M2-type macrophages, slow or improve NEC progression, reduce inflammatory response, and alleviate intestinal damage and histological changes.

### 3.4 Changes of metabolites in intestinal microenvironment after M2NVs treatment

**Figure 6:**
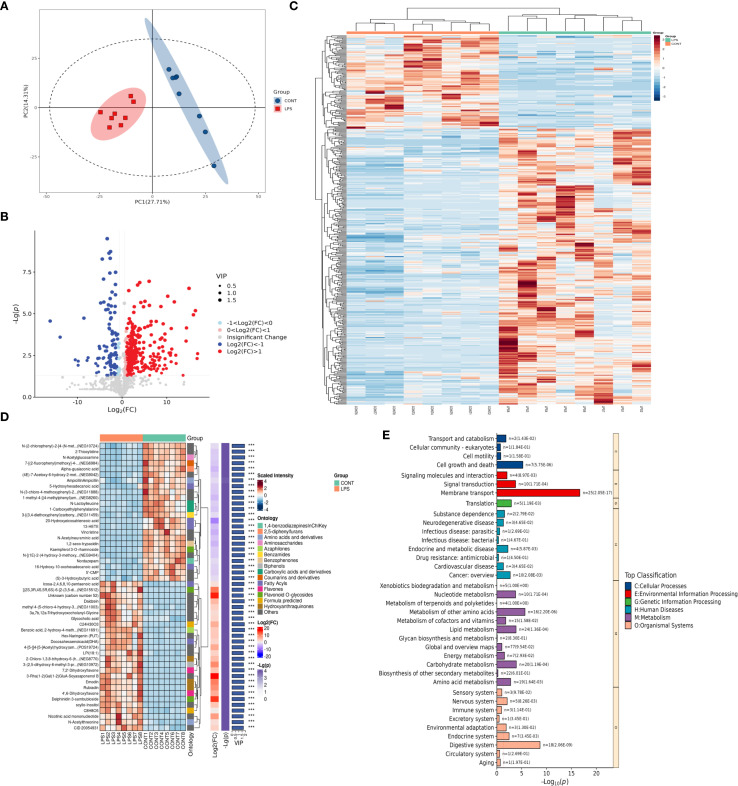
Changes of metabolites in intestinal microenvironment in control and LPS-treated NEC group. **(A)** The quality control analysis was shown in the control and LPS-treated NEC group. **(B)** Volcano plot showing differential metabolites of WT and NEC rats. The red markers represent those that were significantly upregulated, and the green markers represent those that were significantly downregulated, while insignificantly altered are highlighted in gray. **(C)** Hierarchical clustered heatmap of differential metabolites of WT and NEC rats (top 300). **(D)** The complex clustered heatmap of differential metabolites of WT and NEC rats (top 50). **(E)** KEGG enrichment analysis identified the most significantly altered signaling pathways in the control and LPS-treated NEC group.

To analyze the changes in the intestinal microenvironment underlying the biological effects of M2NVs, differential analysis of intestinal metabolites was performed in the intestinal tissues of WT and NEC rats. In our animal model, the variation of the sample concentration within the group is small but the variation between the groups is obvious, indicating that our model is stable, reliable and has better predictability (**Figure 6A**). 423 differentially expressed metabolites (*P.*value < 0.05) were produced into a volcano map to show the up- and down-regulated differential metabolites (**Figure 6B**). Metabolites that had the most significant differences (top 300) associated with microenvironment change were then selected and analyzed in the heatmap and a complex heat map was produced based on these target metabolites (top 50) shown in **Figures 6C, D** and found that there were indeed differences in gut metabolites between normal and NEC rat models. Then, KEGG signaling pathway analyses were carried out on the metabolites with the most obvious differences and found that the membrane transport system and digestive system play an important role in the change of intestinal microenvironment in NEC (**Figure 6E**).

**Figure 7:**
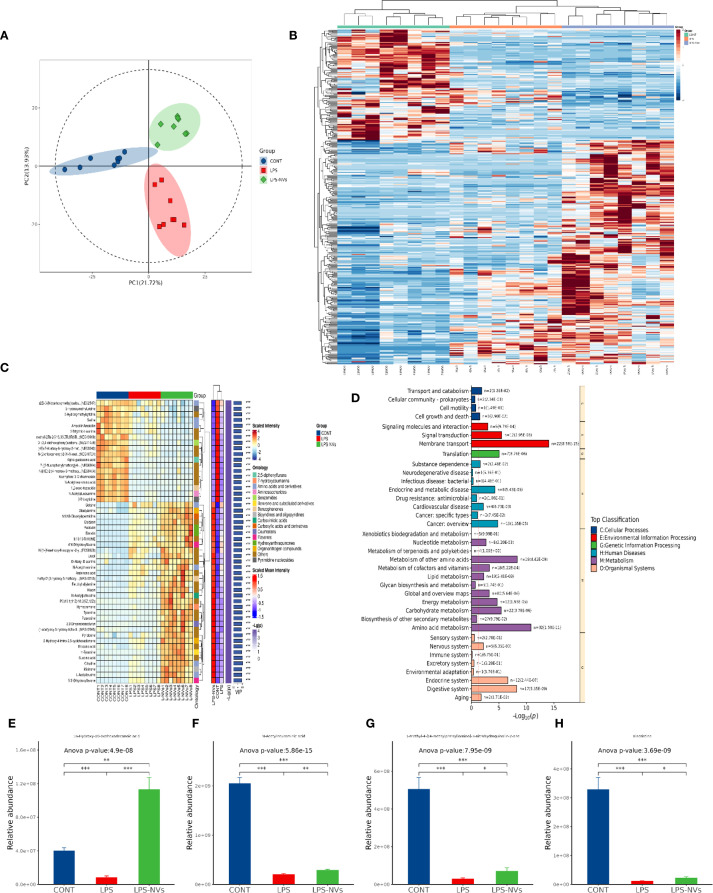
Changes of metabolites in intestinal microenvironment after M2NVs treatment. **(A)** The quality control analysis was shown after M2NVs Treatment. **(B)** Hierarchical clustered heatmap of differential metabolites of WT, NEC and M2NVs treated rats (top 300). **(C)** The complex clustered heatmap of differential metabolites of WT, NEC rats and M2NVs treated rats (top 50). **(D)** KEGG enrichment analysis identified the most significantly altered signaling pathways after M2NVs treatment. **(E-H)** Differential metabolite abundance analysis in intestinal differential metabolites after M2NVs treatment (*p < 0.05, **p < 0.01, ***p<0.001).

Meanwhile, we also screened for differences in metabolites among WT, NEC and M2NVs-treated groups, quality analysis showed that our models are stable and predictive (**Figure 7A**). In **Figures 7B, C** showed that the metabolites that had the most significant differences (top 300) and target metabolites (top 50) associated with microenvironment change were selected and analyzed in the heatmap and complex heatmap and found that there were differences in gut metabolites among these groups. Therewith, KEGG enrichment analysis was performed to identify the most significantly altered signaling pathways after the NEC and M2NVs-treated group, the similarity among these comparisons was the membrane transport system and the digestive system included in the intestinal microenvironment changes (**Figure 7D**). Differential metabolite abundance analysis revealed that 16-Hydroxy-l0-oxohexadecanoic acid, N-Acetylneuraminic acid, 1-methyl-4-[(4-methyl phenyl)amino]-3-nitrohydroquinolin-2-one, Vincristine have significant differences in intestinal differential metabolites (*p* < 0.01, **Figures 7E–H**), which also indicated that M2NVs not only have anti-inflammatory effects but also participate in NEC intestinal environment changes. Generally, the metabolomics analysis results implied that M2NVs intervention could influence the inflammation and membrane transport-related pathways in the intestinal tissues of NEC rats.

## 4 Discussion

The role of inflammatory regulation in the pathogenesis of neonatal NEC has grown increasingly significant. Therefore, we established a continuous extrusion method for collecting and distinguishing M2NVs. Furthermore, we developed an LPS-treated cell model and a neonatal rat model of NEC to investigate the role of M2NVs in NEC treatment. Our results demonstrated that M2NVs could effectively improve NEC-induced intestinal injury and inflammation. The studies on LPS-treated cell injury indicated that M2NVs effectively promoted the transformation of M1-type macrophages into M2-type macrophages and reduced the inflammatory response triggered by LPS. Additionally, in the NEC neonatal rat model experiment, we discovered that M2NVs produced through the continuous extrusion process could foster the polarization of M1 to M2 macrophages and inhibit inflammatory injury *in vivo*, aligning with the *in vitro* results. These findings suggest that M2NVs alleviate NEC-induced inflammatory injury.

Recently, immune cell-derived nanovesicles (NVs) have been extensively investigated, particularly regarding the safety and potential therapeutic effectiveness of immunotherapy. Moreover, the targeted use of NVs has emerged as a strategy to amplify their therapeutic potential (Shin et al., 2024). Macrophages, a key element of intestinal innate immunity, gather and infiltrate the intestinal mucosa, which is regarded as the major pathological feature of NEC and plays a vital role in regulating inflammation, eliminating pathogens, and maintaining dynamic intestinal balance (Wei et al., 2023). Therefore, we analyzed the immunomodulatory function of macrophage NVs produced from macrophages in our experiments. Macrophage polarization and infiltration are widely considered predominant effectors in the experimental NEC model, along with the increased production of proinflammatory mediators, which determine the extent of injury in NEC (Hackam and Sodhi, 2018). As mentioned above, M1 and M2 are opposing phenotypes of macrophages that exhibit different forms and functions. Numerous studies have confirmed that LPS intervention is the conventional stimulus for M1 macrophage polarization, which produces proinflammatory cytokines that further aggravate NEC-associated inflammation (Wen et al., 2020; Liu et al., 2023). In contrast, the M2 phenotype resolves inflammation by producing anti-inflammatory factors. Similarly, our study found that RAW264.7 cells could polarize into spindle-shaped M1 cells with strong adhesion induced by LPS and high expression of the M1 marker CD86, releasing proinflammatory factors such as IL6 and TNFα. Conversely, the M2 phenotype (presented as a prototype with weak adhesion) was polarized by IL4 with high expression of the marker CD206, accompanied by the production of anti-inflammatory factors such as IL4 and TGFβ. In summary, our results confirmed that we successfully established the macrophage polarization model and demonstrated its corresponding characteristics *in vitro*.

Currently, cellular nanotechnology has gradually emerged as a potential therapeutic approach for many diseases. Despite its widespread application, the immunogenicity and potential toxicity of exogenous synthetic nanomaterials may impede further clinical application and commercialization. Consequently, membrane-derived extracellular vesicles (EVs) have been extensively studied due to their advantages of good biocompatibility, suitable size (ranging from 40 nm to 120 nm), and low immunogenicity (Mondal et al., 2023). Macrophages, which are immune cells that combat pathogens and repair injured tissues, have membranes with immune recognition functions and have been formulated into nanovesicles for use in immune diseases, tumors, and other areas (Huang and Zhang, 2024). However, finding alternative methods is particularly urgent due to the limited secretion of exosomes. As is well known, the imbalance between M1 and M2 macrophages contributes to disease pathophysiology, and prior studies have shown that M1 nanovesicles could significantly enhance M2-to-M1 polarization in tumor microenvironment modulation (Wang et al., 2023; Zhou et al., 2024). Therefore, we propose manufacturing M2 nanovesicles to facilitate the transformation of M1 into M2 to reduce inflammation. In this study, we concentrated on the regulatory effect of M2 nanovesicles on the inflammatory reaction in necrotizing enterocolitis (NEC). *In vitro* experiments revealed that the nanovesicles (M0, M1, M2) produced by continuous extrusion exhibit a membrane structure and moderate concentration. Consistent with our expectations, we found that M2 nanovesicle intervention could polarize M1-type macrophages into M2, as evidenced by a decrease in the M1 marker CD86 and a significant increase in the M2 surface marker. Moreover, inflammatory factors were weakened while pro-inflammatory factors increased in both the LPS-induced cell model and the neonatal rat NEC model. Additionally, M2 nanovesicle intervention improved the growth and developmental levels of NEC rats and mitigated the extent of intestinal damage. In conclusion, in NEC, the polarization state of macrophages determines the progression of the disease. M2 nanovesicles play a crucial role in promoting M1 polarization and stimulating intestinal tissue reconstruction and repair. By regulating macrophage polarization, the abilities of phagocytosis and bactericidal activity can be enhanced, subsequently increasing resistance to infections.

Over the past few decades, research on the early prediction and diagnosis biomarkers of NEC has rapidly advanced due to its potentially devastating impact and initial non-specific signs and symptoms. Consequently, “omics” technical analysis has been applied to comprehensively and systematically detect and elucidate the mechanisms of the disease and host-pathogen interactions, thereby improving our understanding of various aspects of NEC-related diseases in premature infants (Ng et al., 2015; Rusconi et al., 2017). Although NEC metabolomics studies have not identified a uniform omics signature, the results indicate that intestinal permeability, energy expenditure, and pathways associated with the inflammatory response may be influenced by the inflammatory state (Moschino et al., 2022). Previous studies have also shown that metabolic profiles differ in premature newborns and NEC infants starting at birth (Sinclair et al., 2020). Thus, we initially investigated whether the M2NVs intervention could impact the types of metabolites and contribute to the search for biomarkers. In our study, we first screened the differential metabolites between normal neonatal rats and the NEC model rats and found that the up- and down-regulated differential metabolites did exist under quality control. After enrichment analysis of the metabolites with obvious differences in our screening, we found that most of the metabolites were closely related to the membrane transport system and the digestive system. Subsequently, we conducted a new round of differential metabolite analysis on the intestinal tissues after M2NVs intervention and found that consistent with the previous enrichment results, these metabolites were also closely related to the membrane transport system and digestive function system, and based on this result, we found that there were significant differences in metabolites such as 16-Hydroxy-l0-oxohexadecanoic acid, N-Acetylneuraminic acid, 1-methyl-4-[(4-methylphenyl)amino]-3-nitrohydroquinolin-2-one, Vincristine, which might provide the potential biomarkers of biological metabolism for our experiment. Collectively, our findings may well explain the M2NVs intervention could not only effectively alleviate the intestinal inflammatory environment, but also affect the changes in intestinal metabolism, so it is reasonable to suppose that targeting manufacture and the polarization role of M2NVs might provide a potential treatment for NEC inflammatory injury.

One limitation of our experiment is the lack of more detailed metabolic screening, varying levels of intestinal damage and intestinal nutrient delivery also affect the differential expression of metabolites. Therefore, future studies will assess the metabolites across various NEC levels to identify stable differentially expressed metabolites after M2NVs intervention. Furthermore, the pathways linking inflammation levels to NEC, including specific metabolites, should be explored in future research. Finally, additional studies are urgently needed to determine the cause or consequence of M2NVs intervention regarding the significant differences associated with metabolic changes before the diagnosis of NEC over time.

## 5 Conclusions

In conclusion, we developed the extraction of exosome-mimicking M2NVs with enhanced anti-inflammatory properties in high yield. Results indicated that M2NVs play a positive role in NEC-induced intestinal injury and inflammation by influencing the polarization of M1-type macrophages toward the M2 phenotype in both *in vivo* and *in vitro* experiments. Furthermore, our designed M2NVs have satisfactory biological properties and effective inflammatory regulatory functions, leading to the favorable treatment of NEC through regulating macrophage polarization, similar to M2 macrophages. We revealed that M2NVs, which exhibited continuous release of anti-inflammatory factors along with excellent mechanical advantages and biological functions, offer a unique combination of three key benefits that make this alternative treatment system more innovative for treating NEC. First, our results showed that the mechanized production of M2NVs can replicate the original functions of macrophage cells and provide a superior yield compared to exosomes. Second, we revealed the comprehensive biological functions of M2NVs through metabolomics analysis, which further elucidates the therapeutic mechanisms of M2NVs and highlights their potential application in NEC treatment. Third, our engineered M2NVs possess broad prospects for clinical application: they are nontoxic, biocompatible, easy to prepare, structurally similar to exosomes, and offer a higher yield than natural exosomes, making M2NVs promising candidates for exosome alternatives. Overall, the engineering strategy of M2NVs represents a promising approach with great potential for NEC treatment.

## Data Availability

The original contributions presented in the study are included in the article/supplementary material. Further inquiries can be directed to the corresponding author.
